# Risk and protective factors for the development of ADHD symptoms in children and adolescents: Results of the longitudinal BELLA study

**DOI:** 10.1371/journal.pone.0214412

**Published:** 2019-03-25

**Authors:** Anne Wüstner, Christiane Otto, Robert Schlack, Heike Hölling, Fionna Klasen, Ulrike Ravens-Sieberer

**Affiliations:** 1 Department of Child and Adolescent Psychiatry, Psychotherapy, and Psychosomatics, University Medical Center Hamburg-Eppendorf, Hamburg, Germany; 2 Department of Epidemiology and Health Monitoring, Robert Koch Institute, Berlin, Germany; Chiba Daigaku, JAPAN

## Abstract

**Background:**

Attention-deficit/hyperactivity disorder (ADHD) is one of the most common neurodevelopmental disorders in childhood worldwide, and causes significant impairments in overall functioning. In order to develop effective prevention and intervention programs, knowledge of the determinants that have an impact on the onset and development of ADHD symptoms is essential. So far, little is known about factors affecting ADHD symptoms in children and adolescents over time. Therefore, this study investigates potential psychosocial risk and protective factors for ADHD symptoms based on cross-sectional and longitudinal data of a German population-based study.

**Methods:**

Data on children and adolescents (*n* = 1,384 aged 11 to 17 years) were collected at three measurement points (baseline, 1-year and 2-year follow-ups) covering a period of two years. We used latent growth modelling to investigate effects of parental mental health problems (risk factor) and self-efficacy, family climate and social support (protective factors) on symptoms of ADHD based on cross-sectional as well as longitudinal data. Sociodemographic factors, pre- and postnatal factors, and comorbid symptoms of internalizing and externalizing mental health problems were considered as covariates.

**Results:**

At baseline, male gender, younger age, stronger aggressive behavior, and stronger parental mental health problems were related to more ADHD symptoms. Longitudinal analyses showed that female gender, migration status, increasing symptoms of generalized anxiety, increasing aggressive behavior and increasing parental mental health problems were associated with stronger increase of ADHD symptoms over time. However, improving family climate was related to decreasing ADHD symptoms over time. We further found moderator effects for social support.

**Conclusion:**

The findings of the study provide important information concerning risk and protective factors in the context of ADHD. Hence, the results may be integrated into the planning and implementation of future prevention and early intervention strategies that target affected children and adolescents.

## Introduction

Attention-deficit/hyperactivity disorder (ADHD) as defined by DSM-5 and ICD-10 is characterized by symptoms of inattention, hyperactivity and/or impulsivity that are present in different settings and cause significant impairments in social and academic functioning [1, 2]. ADHD is considered one of the most common neurodevelopmental disorders in childhood and adolescence worldwide. A meta-analysis including 135 studies found that the global prevalence rate of ADHD is relatively stable over time with an estimated prevalence of about 5% in children and adolescents aged 18 years or younger [3]. Similar prevalence rates were found in a German representative sample of children and adolescents aged 3 to 17 years, with boys being four times more likely to be diagnosed with ADHD than girls [4]. In about 25% of affected children, ADHD is first diagnosed before the age of six [5]. Even though a decrease in symptoms with age can be observed [6], persistence during adolescence and adulthood is high, with approximately two thirds of affected children showing ongoing symptoms [7, 8].

The etiology of ADHD is considered a multifactorial process, whereby the genetic predisposition and biological vulnerabilities are assumed to be most important [9, 10]. Comorbidity in children with ADHD is high, with oppositional defiant and conduct disorders [11, 12], as well as anxiety and depressive disorders [13] being the most common comorbid disorders. Moreover, children with ADHD often experience significant impairments in adolescence and young adulthood. ADHD has a negative impact on academic and educational achievement [14, 15], on social functioning and peer relationships [14, 16] and on family life [17]. Furthermore, experiencing ADHD symptoms has a significant impact on children’s health-related quality of life [18]. Overall, ADHD creates a heavy burden for both individuals and society, resulting in a high relevance for public health.

The summarized findings highlight the importance of effective prevention and intervention programs for children suffering from ADHD in order to prevent the onset of ADHD symptoms and the associated long-term consequences. In this context, knowledge of the determinants that have an impact on the onset and the development of ADHD symptoms over time is essential. Within research on mental health, the investigation of risk and protective factors has become increasingly important in recent years [19]. While risk factors increase the probability of mental health problems, protective factors can strengthen children’s mental health when being exposed to risks [20, 21]. In the literature, risk and protective factors are commonly divided into personal, familial, and social factors [20, 22].

Only few studies have investigated the effects of psychosocial risk and protective factors in the context of ADHD so far. Cross-sectional studies found protective effects of personal factors such as a high sense of coherence [23] and self-efficacy [24] on ADHD in children and adolescents. Self-efficacy is a concept that describes the extent of one’s belief in one’s own competence to reach goals. The protective effect arises from the fact that children with high self-efficacy believe in their personal competence and are therefore better able to cope with stress [25]. Similar findings can be drawn from Dvorsky et al. [26], who examined factors that promote resilience in children with ADHD and found that positive self-perceptions of one’s own competence protect children from negative outcomes of ADHD. With respect to familial resources, positive parenting [26] and a positive family climate [27, 28] have been identified to have a positive impact on children’s ADHD symptoms. Concerning social resources, social support has proven to be a strong protective factor against child mental health problems [29–32] and also in the context of ADHD [27, 28].

In addition, parental mental health problems have demonstrated to be a well-established risk factor for child psychopathology in general [29, 32–35] and in the context of ADHD. In particular, parental ADHD symptoms [36] and maternal depressive symptoms [37–39] have been associated with the development of childhood ADHD. Other familial risk factors include family conflicts [40] and adverse parenting conditions, characterized by a lack of warmth towards the child [36].

Apart from the psychosocial risk and protective factors mentioned above, further studies reported for instance that pre- and postnatal factors such as premature birth, low birth weight and maternal substance use during pregnancy are associated with a higher risk of ADHD [37, 39]. Besides, cross-sectional studies have demonstrated associations of socioeconomic factors such as financial difficulties and low parental education with ADHD symptoms in children and adolescents [37, 41].

The current state of research, however, demonstrates a lack of longitudinal studies on personal, familial, and social determinants for the development of ADHD symptoms. In particular, comparatively little information is available from population-based studies on risk and protective factors affecting the change in children’s and adolescent’s ADHD symptoms over time. Therefore, the current study aims to add to the existing literature by investigating the cross-sectional as well as longitudinal influences of risk and protective factors on the development of childhood ADHD symptoms. Based on the above mentioned findings, we expected that parental psychopathology as risk factor, and self-efficacy, family climate and social support as protective factors not only have an impact on initial ADHD symptoms, but also on the development of ADHD symptoms over time. Since research has shown that risk and protective factors not only have a direct effect on an outcome, but tend to interact in different ways [42], the study also aims to explore potential interaction effects between risk and protective factors over time. It is assumed that the investigated protective factors moderate the detrimental effect of parental mental health problems on ADHD symptoms initially and over time.

## Materials and methods

### Study

The longitudinal BELLA study is the mental health module of the German National Health Interview and Examination Survey for Children and Adolescents (KiGGS) [43]. The BELLA study gathers data on mental health, health-related quality of life, mental health care use, as well as on risk and protective factors for mental health problems for German children, adolescents, and young adults. Baseline assessments for the KiGGS and the BELLA study were both conducted from 2003 to 2006 in close cooperation. The final KiGGS study population included 17,641 children and adolescents aged 0 to 17 years (response rate: 66.6%) [43]. For the BELLA study, a subsample of the KiGGS baseline sample was randomly drawn (*n* = 2,942 children and adolescents aged 7 to 17 years). These children, adolescents and their parents were informed about the BELLA study and asked to participate. Finally, *n* = 2,863 (response rate: 97.3%) children and adolescents (aged 7 to 17 years) and their parents gave their written informed consent and participated in the BELLA baseline assessment. To follow up the baseline sample, further measurement points of the BELLA study were conducted including a 1-year (2004 to 2007) and a 2-year follow-up (2005 to 2008). Of the BELLA baseline participants, *n* = 2,423 (84.6%) participated in the 1-year follow-up and *n* = 2,190 (76.5%) participated in the 2-year follow-up. Detailed descriptions of the KiGGS and the BELLA study have already been published [43, 44]. Data were collected by computer-assisted telephone interviews and subsequent paper-pencil questionnaires. The telephone interviews were conducted by trained interviewers, who followed structured guidelines and were supervised by a child and adolescent psychologist. Self-reported data were gathered from participants aged at least 11 years, parent-reported data were additionally collected from one parent of each participant. Standardized, psychometrically sound and internationally tested measures were administered, if available. The BELLA study was approved by the ethics committee of the University Hospital Charité in Berlin and the Federal Commissioner for Data Protection in Germany. For further details on the design and methods of the longitudinal BELLA study, see Ravens-Sieberer et al. [44] and Klasen et al. [45].

### Participants

In the present study, we analyzed data from the first three measurement points of the BELLA study (baseline, 1-year and 2-year follow-ups) covering a period of 2 years. Participants aged 11 to 17 years at baseline could be included in the analyses, if i) relevant information gathered only at baseline were completely available (on age, gender, socioeconomic status (SES), migration status, premature birth, and maternal smoking and alcohol use during pregnancy) and if ii) longitudinally measured data were available for at least one of the three measurement points (on symptoms of ADHD, parental mental health problems, self-efficacy, family climate, social support, and comorbid symptoms of depression, generalized anxiety, aggressive and dissocial behaviors). Further, cases were only analyzed if the same parent reported on parental mental health problems at each investigated measurement point in the BELLA study. The final sample under analysis included *n* = 1,384 children and adolescents (aged 11 to 17 years at baseline).

### Measures

#### Sociodemographic variables

Age (in years), gender and the SES were determined at baseline. The SES was assessed using the parent-reported Winkler-index [46], which gathers information on education, profession and income of both parents. This index provides a sum-score ranging from 3 to 21, which was used in the following analyses. Only for the purpose of sample description, the sum-score was categorized to create groups of participants with low (scores from 3 to 8), middle (scores from 9 to 14) and high SES (scores from 15 to 21) [47]. Further, we assessed the migration status following Schenk [48]. Migration background was assumed, if i) the child or adolescent had immigrated to Germany and had at least one parent born in a country other than Germany, or if ii) both parents immigrated to Germany or did not hold German citizenship.

#### Pre- and postnatal factors

Parent-reported information on premature birth and maternal substance use (i.e., smoking and alcohol use) during pregnancy were gathered by means of items included in the paper-pencil questionnaire administered to the parents at baseline of the KiGGS study. Concerning premature birth, parents were asked if the child was born full-term (born in the period of three weeks before and two weeks after the predicted birth date), post-maturely or pre-maturely. For the presented analyses, item responses were collapsed resulting in a dichotomous score (0 = “full-term or postmature birth” and 1 = “premature birth”). For maternal substance use during pregnancy, two items (i.e., “Did the mother of the child smoke during pregnancy?” and “Did the mother of the child drink alcohol during pregnancy?”) were presented with three response options each (0 = “not at all”, 1 = “from time to time” and 2 = “regularly”). Responses were collapsed resulting in dichotomous scores for maternal smoking as well as for maternal alcohol use during pregnancy (0 = “not at all” and 1 = “from time to time or regularly”).

#### Symptoms of Attention-deficit/hyperactivity disorder

Symptoms of ADHD in children and adolescents were measured at each investigated measurement point based on a parent-reported Conners Global Index (C-GI) [49, 50]. A German version of the measure was developed and administered in the BELLA study [51–53]. The parent-reported subscale restless-impulsivity of the German version of the C-GI was used in the present analyses, which includes 7 items focusing on ADHD symptoms in children and adolescents such as inattention (e.g., “inattentive, easily distracted”), hyperactivity (“fidgeting”) and impulsivity (“excitable, impulsive”); items were offered with a 4-point response scale (0 = “not true at all” to 3 = “very much true”). We calculated the mean across the administered items with a higher mean indicating more severe symptoms of ADHD. Good internal consistency was found for the C-GI scale restless-impulsivity in the investigated sample (Cronbach’s α ranged from .77 to .82 across measurement points).

#### Comorbid symptoms of depression, generalized anxiety, aggressive and dissocial behaviors

Comorbid symptoms were assessed at each investigated measurement point. Depressive symptoms in the children and adolescents were measured using the self-reported German version of the established Center for Epidemiologic Studies Depression Scale (CES-DC) [54, 55]. By means of 20 items, the CES-DC gathers emotional, cognitive and behavioral aspects of depression (e.g., “I felt that everything I did was an effort”); items are presented with a 4-point response scale (0 = “not at all” to 3 = “a lot”). The mean across all CES-DC items was calculated with a higher mean indicating more severe depressive symptoms. The internal consistency for the CES-DC was good in the investigated sample (Cronbach’s α ranged from .83 to .88 across measurement points).

Self-reported symptoms of generalized anxiety in children and adolescents were assessed based on a German version of the Screen for Child Anxiety Related Disorders (SCARED-D) [56–58]. The scale on generalized anxiety of the SCARED-D was administered including 9 items (e.g., “I worry about what is going to happen in the future”) offered with a 3-point response scale (0 = “not true or hardly ever true” to 2 = “very true or often true”). We calculated the mean across the SCARED-D items with a higher mean indicating more severe symptoms of generalized anxiety. The internal consistency of the administered scale of the SCARED-D was good in the investigated sample (Cronbach’s α ranged from .81 to .85).

Parent-reported aggressive and dissocial behaviors in children and adolescents were assessed based on the German version of the well-established Child Behavior Checklist (CBCL) [59, 60]. This measure provides a scale on externalizing problems including the subscales aggressive behavior with 20 items (e.g., “Behavior of your child: Attacks others”) and dissocial behavior with 13 items (e.g., “Behavior of your child: Steals at home”). CBCL items are offered with three response options (0 = “not true” to 2 = “very true or often true”). We calculated the mean across the items for each subscale with higher means indicating more severe aggressive and dissocial behaviors. Good to excellent internal consistency was found for the CBCL subscale aggressive behavior (Cronbach’s α ranged from .88 to .90) and acceptable to good internal consistency was given for the subscale on dissocial behavior in the investigated sample (α ranged from .69 to .75).

#### Risk factor parental mental health problems

The Symptom-Check List 9-item Short version (SCL-S-9) [61] served to assess parental mental health problems reported by one parent of each participant at each investigated measurement point of the BELLA study. The SCL-S-9 is an abbreviated version of the SCL-90-R [62] assessing a wide range of psychopathologic symptoms by means of 9 items. Each item of the SCL-S-9 belongs to one dimension of the original SCL-90-R (i.e., somatization, obsessive-compulsive, interpersonal sensitivity, depression, anxiety, hostility, phobic anxiety, paranoid ideation, and psychoticism) and is presented with a 5-point response scale (0 = “none at all” to 4 = “very severe”). The mean across all SCL-S-9 items was calculated (i.e., the Global Severity Index); a higher mean indicates more severe psychopathologic symptoms. Good internal consistency was given for the SCL-S-9 in the investigated sample (Cronbach’s α ranged from .81 to .84 across measurement points).

#### Protective factors self-efficacy, family climate and social support

Protective factors were measured at each investigated measurement point. To measure self-reported self-efficacy in children and adolescents, the General Self-Efficacy Scale (GSE) [63, 64] was used. The GSE includes 10 items (e.g., “I can usually handle whatever comes my way”) offered with a 4-point response scale (0 = “not at all true” to 3 = “exactly true”). The mean across all GSE items was calculated with a higher mean indicating higher self-efficacy. The internal consistency was good for the GSE in the investigated sample (Cronbach’s α ranged from .78 to .83 across measurement points).

The family climate was assessed in children and adolescents based on the German Family Climate Scale (FCS) [65]. The FCS is the German adaptation of the Family Environment Scale (FES) [66]. In the BELLA study, 8 items of the FCS related to active recreational organization and cohesion (e.g., “In our family everybody cares about each other’s worries”) were administered. Items were presented with a 4-point response scale (0 = “not true” to 3 = “exactly true”). The mean across the 8 FCS items was calculated with a higher score indicating a better family climate. Good internal consistency was found for the administered FCS in the investigated sample (Cronbach’s α ranged from .78 to .83 across measurement points).

Self-reported social support in children and adolescents was measured by means of the German translation of the Social Support Survey (SSS) [67]. Items of the original SSS that were not applicable for children and adolescents were excluded from the BELLA study and the wording of some items was slightly modified. The administered SSS-short included 8 items assessing how frequent specific types of support were available (e.g., “How often is the following type of support available for you if you need it? Someone you can count on to listen to you, when you need to talk”). Items were offered with a 5-point response scale (0 = “none of the time” to 4 = “all of the time”). The mean across the 8 SSS-short items was calculated with a higher score indicating more available social support. The internal consistency was good to excellent for the SSS-short in the investigated sample (Cronbach’s α ranged from .88 to .91 across measurement points).

### Data analysis

We used latent growth modelling to analyze our cross-sectional and longitudinal data. This analyzing approach is used in social, psychological and health research frequently; it is especially recommended for analyses of change in behavior [68]. By means of a latent growth model (LGM), two latent factors can be calculated (i.e., intercept and slope) using a regression-type line of the variable under investigation over time. The intercept represents the initial status of the variable at baseline and the slope reflects the change in the variable over time.

In the present study, data analyses followed a two-step procedure. At first, we calculated an LGM for each longitudinally measured construct under analyses (i.e., for symptoms of ADHD, for the risk factor parental mental health problems, for protective factors self-efficacy, family climate and social support, and for comorbid symptoms of depression, generalized anxiety, aggressive and dissocial behaviors). Subsequently, we used intercepts and slopes resulting from LGMs in linear regression models. Regression Model A0 served to investigate whether initial symptoms of ADHD were predicted by initial parental mental health, self-efficacy, family climate and social support. Regression Model B0 was used to analyze if the change in ADHD symptoms was predicted by the initial state of parental mental health, self-efficacy, family climate and social support, as well as by the change in these variables over time. The following covariates were added to Models A0 and B0: i) sociodemographic variables (i.e., age, gender, SES, and migration status), ii) information on premature birth, and maternal smoking and alcohol use during pregnancy, and iii) comorbid symptoms of depression, generalized anxiety, aggressive and dissocial behaviors (we added corresponding intercepts to Model A0, and intercepts and slopes to Model B0). For each regression model, all included variables were entered simultaneously into the model.

Moreover, we aimed to explore whether potentially protective factors moderate the relationship between the risk factor parental mental health and ADHD symptoms in the children and adolescents. For this purpose, we additionally included interaction terms in our linear regression models. We thus examined if the association between initial ADHD symptoms and initial parental mental health problems was moderated by initial self-efficacy, initial family climate or initial social support (Model A1). Additionally, we investigated whether the associations between the change in ADHD symptoms and the initial state of parental mental health as well as the change in parental mental health were moderated by self-efficacy, family climate or social support (Model B1).

For all regression models, we centered metric variables. To evaluate the strengths of detected effects, we followed Cohen’s rules of thumb [69]: a standardized regression weight (β) of .1 indicates a weak, β of .3 reflects a medium and β of .5 points out a strong effect. We used Mplus 8 [70] for calculating LGMs and IBM SPSS 22 for regression analyses.

## Results

The analyzed sample including *n* = 1,384 children and adolescents aged 11 to 17 years at baseline is described in Table 1. About half of the investigated children and adolescents were female, the mean age was about 14 years, about half of the participants lived in families with a medium SES (low SES: 22%, *n* = 306; medium SES: 51%, *n* = 706; high SES: 27%, *n* = 372), and 4% of the analyzed children and adolescents had a migration status. Questions on parental mental health problems for each investigated measurement point were answered by the mothers of 92% of the participating children and adolescents (*n* = 1,274), by the fathers of 7% (*n* = 103) and by step-, foster- or grandparents for 1% (*n* = 7) of the participants. Concerning items on premature birth and maternal smoking and alcohol use (administered at baseline in the KiGGS study), for 89% (*n* = 1,233) of the participants the mothers, for 8% (*n* = 107) the fathers, and for 3% (*n* = 37) mother and father together responded (for *n* = 3 cases foster-/adoptive- or grandparents responded and for *n* = 4 cases information was missing).

**Table 1:** Description of the analyzed sample of children and adolescents aged 11 to 17 years (at baseline).

	Baseline		1-year follow-up		2-year follow-up	
	*n (%)*	*M (SD)*	*n*	*M (SD)*	*n*	*M (SD)*
**Sociodemographic data**^1^						
Female	706 (51%)					
Age (in years)		13.89 (1.991)				
Socioeconomic status		11.86 (4.106)				
Migration background	49 (4%)					
**Pre- and postnatal factors**^1^						
Premature birth	148 (11%)					
Maternal smoking during pregnancy	209 (15%)					
Maternal alcohol use during pregnancy	206 (15%)					
**Symptoms of ADHD**	1,369	0.68 (0.516)	1,100	0.59 (0.492)	1,086	0.55 (0.467)
**Comorbid mental health problems**						
Depressive symptoms	1,363	0.49 (0.340)	1,068	0.46 (0.342)	1,045	0.45 (0.361)
Symptoms of generalized anxiety	1,363	0.63 (0.377)	1,067	0.60 (0.394)	1,045	0.60 (0.405)
Aggressive behavior	1,305	0.31 (0.271)	1,110	0.28 (0.252)	947	0.27 (0.264)
Dissocial behavior	1,305	0.14 (0.180)	1,111	0.14 (0.166)	947	0.14 (0.182)
**Risk factor**						
Parental mental health	1,369	0.59 (0.510)	1,100	0.58 (0.516)	1,086	0.49 (0.491)
**Protective factors**						
Self-efficacy	1,363	2.14 (0.377)	1,066	2.16 (0.433)	1,045	2.17 (0.400)
Family climate	1,371	1.83 (0.528)	1,085	1.83 (0.524)	914	1.8 (0.528)
Social support	1,365	3.12 (0.734)	1,090	3.29 (0.669)	918	3.32 (0.642)

^1^Sociodemographic data and data on pre- and postnatal factors were available for the complete sample under analysis (n = 1,384).

ADHD = Attention-deficit/hyperactivity disorder.

*M* = mean, *SD* = standard deviation; for measures see text (Methods).

Correlations between the score of the C-GI scale restless-impulsivity and the single items of the SCL-S-9 ranged from .15 to .29 based on baseline data. Small associations were found for psychoticism (r = .15), somatization (r = .16), phobic anxiety (r = .16), paranoid ideation (r = .16), obsessive-compulsive (r = .22), and anxiety (r = .24); nearly moderate associations were detected for hostility (r = .25), interpersonal sensitivity (r = .28), and depression (r = .29).

Results for Model A0 (see Table 2) based on cross-sectional data showed that male gender and younger age were both associated with stronger symptoms of ADHD. Further, stronger aggressive behavior was related to more ADHD symptoms. In addition, strong parental mental health problems were associated with stronger ADHD symptoms in the child. Effects of age, gender and parental mental health problems on ADHD symptoms were small, but we found a strong effect for aggressive behavior on ADHD symptoms.

**Table 2:** Predictors of the initial state and the change of symptoms of attention-deficit/hyperactivity disorder in children and adolescents.

	Regression Model A0^1^ predicting initial symptoms of ADHD			Regression Model B0^2^ predicting change in symptoms of ADHD		
	*b*	β	*p*	*b*	β	*p*
*Constant*	*0*.*72*		< .*001*	*-0*.*07*		< .*001*
**Sociodemographic data**						
Female	-0.10	-.13	< .001	0.01	.08	.007
Age (in years at baseline)	-0.03	-.12	< .001	0.00	-.03	.471
Age by gender	0.00	.00	.985	0.00	.00	.994
Socioeconomic status (at baseline)	0.00	-.03	.101	0.00	.00	.896
Migration background	-0.07	-.03	.102	0.03	.07	.005
**Pre- and postnatal factors**						
Premature birth	0.03	.02	.246	0.00	-.02	.465
Maternal smoking during pregnancy	0.00	.00	.903	0.00	.01	.708
Maternal alcohol use during pregnancy	0.02	.02	.391	0.00	-.01	.604
**Comorbid mental health problems**						
Initial depressive symptoms (intercept)	0.03	.02	.534	0.01	.03	.419
Change in depressive symptoms (slope)				-0.05	-.03	.342
Initial symptoms of generalized anxiety (intercept)	0.04	.03	.213	-0.01	-.04	.258
Change in symptoms of generalized anxiety (slope)				0.05	.06	.049
Initial aggressive behavior (intercept)	1.12	.61	< .001	0.03	.09	.084
Change in aggressive behavior (slope)				2.44	.23	< .001
Initial dissocial behavior (intercept)	-0.02	-.01	.814	-0.01	-.02	.577
Change in dissocial behavior (slope)				0.07	.02	.594
**Risk factor**						
Initial parental mental health (intercept)	0.16	.15	< .001	0.01	.03	.425
Change in parental mental health (slope)				0.28	.14	< .001
**Protective factors**						
Initial self-efficacy (intercept)	-0.06	-.04	.073	0.01	.02	.521
Change in self-efficacy (slope)				-0.01	-.01	.669
Initial family climate (intercept)	0.00	.00	.866	-0.01	-.03	.326
Change in family climate (slope)				-0.04	-.07	.010
Initial social support (intercept)	0.03	.03	.239	0.00	.00	.953
Change in social support (slope)				0.02	.03	.231

For each regression model, all included variables were entered simultaneously into the model.

^1^Linear regression Model A0 (n = 1,384); model fit: adjusted R^2^ = .52; F = 95.14.

^2^Linear regression Model B0 (n = 1,384); model fit: adjusted R^2^ = .07; F = 5.40.

ADHD = Attention-deficit/hyperactivity disorder.

*b* = unstandardized regression coefficient; β = standardized regression coefficient; for measures see text (Methods).

**Fig 1:**
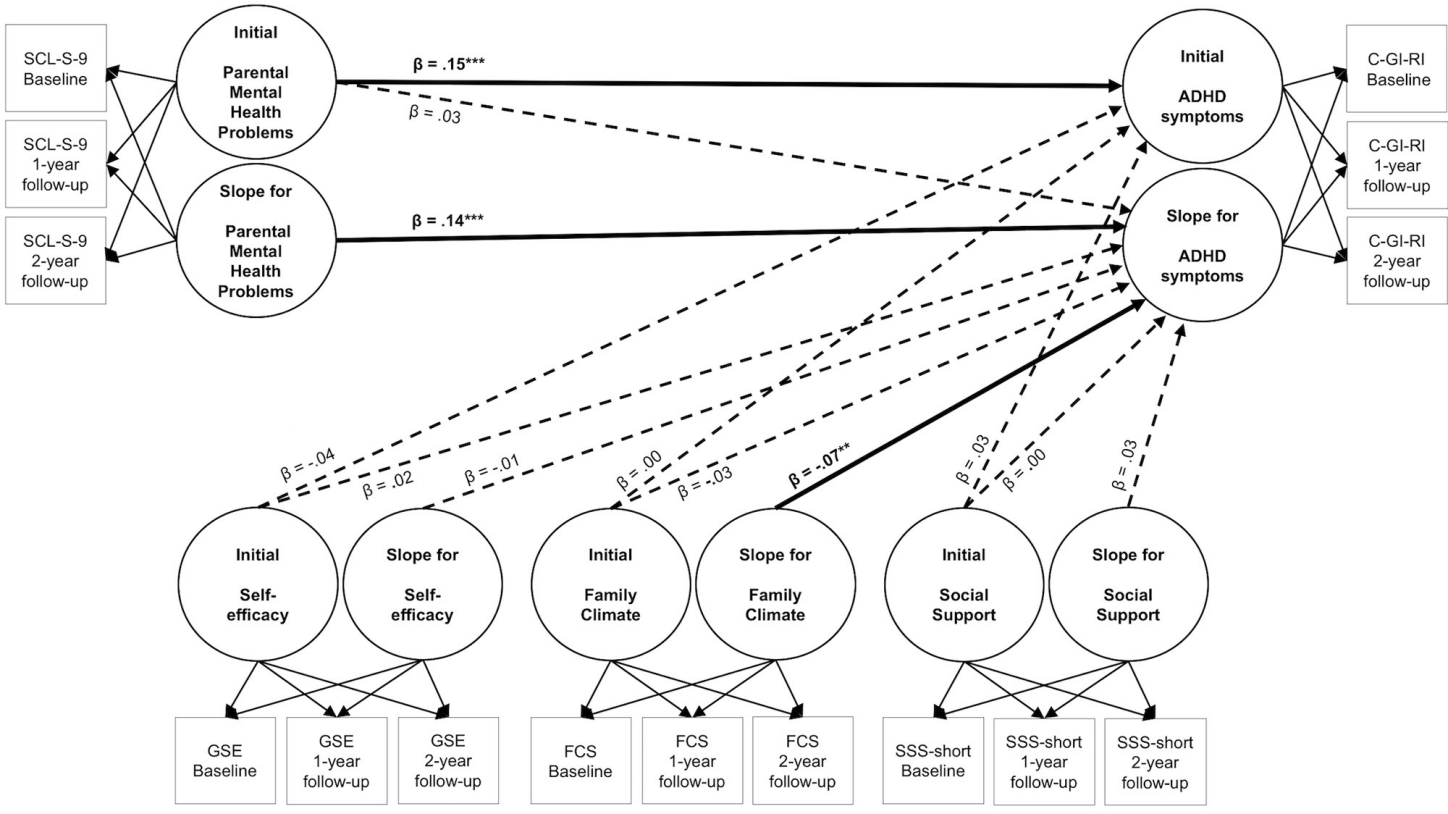
Effects of risk and protective factors on symptoms of attention-deficit/hyperactivity disorder in children and adolescents. Continuous lines mark significant effects, interrupted lines indicate non-significant effects, resulting from regression Models A0 and B0. n = 1,384. ADHD = attention-deficit/hyperactivity disorder; C-GI-RI = subscale restless-impulsivity of the Conners Global Index [49, 50]; SCL-S-9 = Symptom-Check List Short version-9 [61]; GSE = General Self-Efficacy Scale [63, 64]; FCS = an eight-item score based on the Family Climate Scale [65]; SSS-short = eight items of the German version of the Social Support Survey [67]; β = standardized regression coefficient; reported βs are resulting from Models A0 and B0; ***p≤001; **p≤01;

Based on longitudinal data, results for Model B0 (Table 2) showed for girls compared to boys as well as for participants with migration status compared to those without this status a stronger increase in symptoms of ADHD over time. Further, an increase in symptoms of generalized anxiety and increasing aggressive behavior over time were both associated with increasing ADHD symptoms over time. Additionally, increasing parental mental health problems (risk factor) were related to increasing ADHD symptoms over time. In contrast, an improvement in family climate (protective factor) over time was associated with a decrease in ADHD symptoms over time. We found only small effects by means of Model B0. Results of Models A0 and B0 on effects of risk and protective factors on symptoms of ADHD are gathered and graphically presented in Fig 1.

Further regression models were conducted to explore moderator effects of potentially protective factors on the relationship between parental mental health problems (risk factor) and ADHD symptoms. Results are provided as Supplementary Information (Table A in S1 File). Based on baseline data, we found no moderating effects for any investigated protective factor (Model A1). By means of longitudinal data, we detected moderating effects for social support, but not for self-efficacy and family climate (Model B1). Increasing social support over time attenuated the association between increasing parental mental health problems and increasing ADHD symptoms over time. Moreover, we unexpectedly found an association between increasing ADHD symptoms and increasing social support over time only in children of parents with less severe mental health problems. Detected interaction effects were small.

## Discussion

The present study investigated the effects of potential risk and protective factors on the symptoms of ADHD in children and adolescents based on cross-sectional and longitudinal data. As expected, we found that stronger parental mental health problems were associated with more ADHD symptoms at baseline. Additionally, increasing parental mental health problems were associated with increasing ADHD symptoms over time. Contrary to our hypothesis, we found no associations between the protective factors and ADHD initially. However, an improvement in family climate was associated with decreasing ADHD symptoms over time. We further detected moderating effects of social support on the relationship between parental psychopathology and ADHD symptoms over time. Moreover, as assumed, male gender, younger age and comorbid aggressive behaviour were associated with more ADHD symptoms at baseline.

In our baseline analysis, we found that stronger parental mental health problems (risk factor) were associated with more ADHD symptoms in children and adolescents. This result is in line with previous studies on parental psychopathology and child mental health [29, 32–35] as well as with research that points to a strong specific association of parental ADHD with child ADHD problems [71]. Moreover, in our longitudinal analysis, an increase in parental mental health problems was associated with increasing ADHD symptoms over time. The finding that parental psychopathology and ADHD symptoms are associated initially as well as in their development over time, underlines the importance to consider parental mental health in targeted interventions in children and adolescents. Particularly in case of a parental ADHD symptomatology, combined treatments for parents and their child have shown to be successful [72, 73].

Contrary to our assumption and to former research [24, 27, 28], the protective factors self-efficacy, family climate and social support did not predict initial symptoms of ADHD in our study. This might be due to the fact that ADHD is a highly heritable disorder and thus, a remarkable proportion of the variance in ADHD symptoms in our study may has been explained by genetic influences. It is estimated that environmental factors only account for 10 to 40% of the variance associated with ADHD [74]. However, in our longitudinal model, an improvement in family climate over time was associated with a decrease in ADHD symptoms over time. Consequently, our results support the approach of family-based interventions that address dysfunctional family processes and increase mutual support and communication. Such family-based interventions have proven to be effective in treating children and adolescents with ADHD symptoms [75–77].

Moreover, although we could not find any direct effects of the protective factors self-efficacy and social support on ADHD symptoms in our cross-sectional and longitudinal analyses, we detected moderating effects for social support on the relationship between parental mental health problems and children’s and adolescent’s ADHD. Increasing social support over time could attenuate the association between strong parental mental health problems and strong ADHD symptoms. Corresponding moderator effects were found by Klasen et al. [29] in their study on depressive symptoms based on the same longitudinal data set. This finding has important implications for prevention and intervention programs. Strengthening resources such as the availability of good social support in children and adolescents with a mentally ill parent is particularly important to support children and adolescents in coping with the mental illness of their parent. Thus, it is assumed that children of mentally ill parents may benefit particularly from cognitive-behavioral therapies (CBT) that focus on enhancing personal resources such as social skills in order to support positive peer relationships and friendships. The effectiveness of CBT for children and adolescents with ADHD has been widely researched and scientifically proven [78, 79]. Besides, a recent review and meta-analysis found that peer inclusion interventions are effective in enhancing social functioning in children and adolescents with ADHD [80].

In our moderator model based on longitudinal data, we further unexpectedly found an association between increasing ADHD symptoms and increasing social support over time only in children of parents with less severe mental health problems. This finding may at least partly reflect the direct and rather immediate supportive reaction of a healthy social environment on ADHD symptoms in children and adolescents.

Rose et al. [81] suggested a difference between protective factors and resources. Following this approach, family climate should be described as a resource factor based on our results, as we found a direct beneficial effect of family climate on child and adolescent ADHD symptoms. In contrast, social support can be described as protective factor based on our findings, as it moderates the relationship between the risk factor parental mental health problems and ADHD symptoms. Future studies may wish to further investigate other potential kinds of relationships between risk and protective factors and ADHD.

Based on baseline analysis, we further detected that male gender and younger age were associated with stronger symptoms of ADHD in line with results from previous population-based studies [5, 6]. This finding highlights the need for early prevention and intervention programs to be gender-sensitive. Unlike previous studies that reported that low parental education and financial difficulties predict more or stronger ADHD symptoms [37, 41], we did not find associations between SES and symptoms of ADHD. We further found no associations between premature birth, maternal smoking and alcohol use during pregnancy, although previous studies identified pre- and postnatal factors as important predictors of ADHD [37, 39]. However, the role of prenatal factors for the development of ADHD symptoms is somewhat ambiguous. While many studies found a roughly doubled risk for maternal smoking [82, 83], other studies did neither confirm maternal smoking nor the consumption of alcohol in pregnancy as a risk factor for the incidence of ADHD in the child [84, 85]. It should, however, also be noted that items on substance use may have been answered differently in this study, depending on who completed the questionnaire (mother, father or mother and father together). In addition, social desirability may have prevented the parents from reporting on smoking and alcohol use. Future studies may assess and analyze effects of pre- and postnatal factors more detailed.

Regarding the examined comorbid mental health problems, we found a strong association between externalizing symptoms of aggressive behavior and ADHD symptoms, confirming results from previous studies investigating this relationship [11, 12]. Yet, we did not detect any effects of comorbid internalizing symptoms of depression and anxiety at baseline, although it is well known from the literature that ADHD often co-occurs with these disorders [13]. This might be due to the fact that our analyses were based on a population-based sample in which the prevalence of depression and anxiety is generally lower compared to prevalences in clinical samples. However, in our longitudinal model, we found that increasing symptoms of generalized anxiety were associated with increasing ADHD symptoms over time, which coincides with results of former research on the co-occurrence of ADHD and anxiety [13].

Based on the analysis of longitudinal data, we further found that the increase of ADHD symptoms over time was more pronounced in girls compared to boys, which could be explained by the fact that girls are faced with different challenges during the transition from childhood and adolescence to young adulthood compared to boys. Moreover, children and adolescents with migration background also experienced a stronger increase of ADHD symptoms over time compared to youths without migration background. This is of particular concern for prevention and intervention efforts in migrant populations since data from the nationally representative KiGGS baseline study suggest that there might be migrant-specific barriers to the use of health care services, in particular for families with children and adolescents with ADHD symptoms [86]. Further, although previous studies have shown that ADHD symptoms decrease with age [6], no effect of age on the change of ADHD symptoms over time could be found in the present study, which may partly be explained by the fact that our study only covered a period of two years.

The present study has the following limitations. By means of the variables included in our baseline model, a proportion of 52% of the variance in ADHD symptoms was explained. However, we could only explain 7% of the variance in the slope of ADHD symptoms by the variables included in our longitudinal model. Further, we detected only small effects in our longitudinal model, if effects were given at all. This may be due to the fact that we investigated a general population sample with consistently rather low levels of mental health problems, and with rather good self-efficacy, family climate, and social support. Further, our study only covered a period of two years. This issue is reflected in the fact that the slopes for some investigated constructs did not vary significantly across individuals (e.g., for depressive symptoms and for symptoms of dissocial behavior). Future research may cover a longer period of time. However, these findings may as well indicate that the development of ADHD symptoms is associated with important factors that we did not consider in our model based on longitudinal data. These factors may include genetic risks [9, 10], adverse childhood experiences such as physical or sexual abuse [87–89], other pre- and postnatal risks such as low birth weight and young maternal age at birth [39] as well as other personal resources such as resilience [90] and social competence [28]. Apart from this, it would have been interesting to include the use of medication as control variable in our analyses. Future studies on the development of ADHD symptoms may take these aspects into account. Further, it should be noted that the BELLA study is an observational study that only identifies associations between risk and protective factors and ADHD symptoms. In order to investigate cause-effect relationships, other studies such as intervention studies would have to be performed. In addition, ADHD symptoms were assessed with a brief questionnaire in the present study. We chose to analyze the metric scale scores in order to provide information interesting for planning early interventions on systematic associations between risk and protective factors and ADHD symptoms without loss of information (due to potential categorization). However, it is still a limitation of our study, that we were not able to investigate clinical diagnoses on ADHD. Future clinical studies may wish to analyze associations with clinical ADHD diagnoses. Furthermore, our sample only included children and adolescents aged 11 to 17 years at baseline. In view of the fact that in about 25% of affected children, ADHD is first diagnosed before the age of six [5], future studies may also include younger children.

Our study has several strengths. Data was derived from the BELLA study, which is the first comprehensive longitudinal study to assess the mental health and well-being of children and adolescents in Germany. The strengths of the BELLA study include the large population-based cohort and the wide age range of the participants. Further, risk and protective factors were assessed using established measures. Self-reported data of children and adolescents was used to assess the protective factors and comorbid symptoms of internalizing problems. Parent-reported data was used to assess parental psychopathology, symptoms of ADHD and comorbid aggressive and dissocial behaviors since research has shown that externalizing disorders are better observable by parents [91, 92]. Moreover, we used an appropriate analyzing approach that allowed us to analyze changes in ADHD symptoms as well as changes in risk and protective factors over time. Lastly, we included pre- and postnatal factors as well as common comorbidities of ADHD as important covariates in our models.

To the best of our knowledge, this is one of the first studies to investigate the effects of personal, familial and social risk and protective factors on ADHD symptoms in children and adolescents over time. The results of our study indicate that parental mental health problems can have detrimental effects on ADHD symptoms, while a good family climate and social support can have beneficial effects on ADHD in children and adolescents over time.

Given that ADHD is highly prevalent and causes significant impairments in almost all areas of life, our findings have important implications for prevention and clinical practice. Besides family-based interventions, future prevention and early intervention programs should focus on supporting the availability of good social support and on enhancing social skills, particularly in children of mentally ill parents, in order to reduce risks and prevent the onset of ADHD symptoms.

## Supporting information

S1 File Moderator models. S1A Table. Protective factors self-efficacy, family climate, and social support moderating the relationship between parental mental health problems and symptoms of attention-deficit/hyperactivity disorder in children and adolescents.
(DOCX)

S2 File Dataset. (XLS)
