# Supplementary material for: Risk and protective factors for the development of ADHD symptoms in children and adolescents: Results of the longitudinal BELLA study
Source: PLoS One. 2019 Mar 25;14(3):e0214412. doi: 10.1371/journal.pone.0214412 (PMC6433344; doi:10.1371/journal.pone.0214412)
Supplement: S1 File — S1A Table. Protective factors self-efficacy, family climate, and social support moderating the relationship between parental mental health problems and symptoms of attention-deficit/hyperactivity disorder in children and adolescents. (DOCX) [file pone.0214412.s001.docx]

**Supporting Information. S1 File. Moderator models.**

Manuscript title:

**Risk and protective factors for the development of ADHD symptoms in children and adolescents: Results of the longitudinal BELLA study**

Journal:

PLOS One

Authors:

Anne Wüstner^*^, Christiane Otto^1^, Robert Schlack^2^, Heike Hölling, Fionna Klasen^1^, Ulrike Ravens-Sieberer^1*^

^1^ Department of Child and Adolescent Psychiatry, Psychotherapy, and Psychosomatics, University Medical Center Hamburg-Eppendorf, Hamburg, Germany

^2^ Department of Epidemiology and Health Monitoring, Robert Koch Institute, Berlin, Germany

* Corresponding author

E-mail: a.wuestner@uke.de (AW), ravens-sieberer@uke.de (URS)

**S1A Table. Protective factors self-efficacy, family climate, and social support moderating the relationship between parental mental health problems and symptoms of attention-deficit/hyperactivity disorder in children and adolescents.**

|  | **Regression Model A1^1^**  **predicting initial symptoms of ADHD** | | | **Regression Model B1^2^**  **predicting change in symptoms of ADHD** | | |
| --- | --- | --- | --- | --- | --- | --- |
|  | ***b*** | **β** | ***p*** | ***b*** | **β** | ***p*** |
| *Constant* | *0.72* |  | *<.001* | *-0.07* |  | *<.001* |
| **Sociodemographic data** |  |  |  |  |  |  |
| Female | -0.11 | -.13 | <.001 | 0.01 | .08 | .008 |
| Age (in years at baseline) | -0.03 | -.12 | <.001 | -0.00 | -.04 | .381 |
| Age by gender | 0.00 | -.00 | .982 | 0.00 | .01 | .874 |
| Socioeconomic status (at baseline) | -0.00 | -.03 | .110 | -0.02 | -.00 | .966 |
| Migration background | -0.07 | -.03 | .108 | 0.03 | .08 | .003 |
| **Pre- and postnatal factors** |  |  |  |  |  |  |
| Premature birth | 0.03 | .02 | .269 | -0.00 | -.02 | .504 |
| Maternal smoking during pregnancy | -0.00 | -.00 | .892 | 0.00 | .02 | .565 |
| Maternal alcohol use during pregnancy | 0.02 | .02 | .405 | -0.00 | -.02 | .483 |
| **Comorbid mental health problems** |  |  |  |  |  |  |
| Initial depressive symptoms (intercept) | 0.03 | .02 | .551 | 0.01 | .02 | .540 |
| Change in depressive symptoms (slope) |  |  |  | -0.07 | -.04 | .232 |
| Initial symptoms of generalized anxiety (intercept) | 0.05 | .03 | .200 | -0.01 | -.03 | .363 |
| Change in symptoms of generalized anxiety (slope) |  |  |  | 0.05 | .06 | .038 |
| Initial aggressive behavior (intercept) | 1.12 | .61 | <.001 | 0.02 | .07 | .176 |
| Change in aggressive behavior (slope) |  |  |  | 2.33 | .22 | <.001 |
| Initial dissocial behavior (intercept) | -0.02 | -.01 | .836 | -0.01 | -.02 | .703 |
| Change in dissocial behavior (slope) |  |  |  | 0.07 | .02 | .584 |
| **Risk factor** |  |  |  |  |  |  |
| Initial parental mental health problems (intercept) | 0.16 | .15 | <.001 | 0.00 | .02 | .525 |
| Change in parental mental health problems (slope) |  |  |  | 0.26 | .13 | <.001 |
| **Protective factors** |  |  |  |  |  |  |
| Initial self-efficacy (intercept) | -0.06 | -.04 | .070 | 0.01 | .02 | .549 |
| Change in self-efficacy (slope) |  |  |  | -0.01 | -.02 | .504 |
| Initial family climate (intercept) | -0.01 | -.01 | .809 | -0.01 | -.04 | .241 |
| Change in family climate (slope) |  |  |  | -0.04 | -.07 | .009 |
| Initial social support (intercept) | 0.03 | .03 | .219 | 0.00 | .01 | .677 |
| Change in social support (slope) |  |  |  | 0.03 | .05 | .106 |
| **Interactions between the risk and protective factors** |  |  |  |  |  |  |
| Initial parental mental health problems by initial self-efficacy | -0.07 | -.02 | .391 | 0.02 | .03 | .501 |
| Initial parental mental health problems by change in self-efficacy |  |  |  | -0.09 | -.06 | .116 |
| Change in parental mental health problems by initial self-efficacy |  |  |  | 0.30 | .04 | .313 |
| Change in parental mental health problems by change in self-efficacy |  |  |  | -0.05 | -.00 | .940 |
| Initial parental mental health problems by initial family climate | -0.03 | -.01 | .662 | -0.01 | -.01 | .818 |
| Initial parental mental health problems by change in family climate |  |  |  | 0.05 | .03 | .353 |
| Change in parental mental health problems by initial family climate |  |  |  | -0.27 | -.05 | .216 |
| Change in parental mental health problems by change in family climate |  |  |  | -0.62 | -.04 | .250 |
| Initial parental mental health problems by initial social support | -0.01 | -.00 | .847 | -0.02 | -.04 | .336 |
| Initial parental mental health problems by change in social support |  |  |  | -0.13 | -.09 | .012 |
| Change in parental mental health problems by initial social support |  |  |  | 0.18 | .04 | .352 |
| Change in parental mental health problems by change in social support |  |  |  | -1.23 | -.08 | .036 |

^1^Linear regression Model A1 (n = 1,384); model fit: adjusted R^2^ = .52; F = 80.13.

^1^Linear regression Model B1 (n = 1,384); model fit: adjusted R^2^ = .08; F = 4.30.

ADHD = Attention-deficit/hyperactivity disorder.

*b* = unstandardized regression coefficient; β = standardized regression coefficient; for measures see text (Methods).
